# Deep learning of ECG waveforms for diagnosis of heart failure with a reduced left ventricular ejection fraction

**DOI:** 10.1038/s41598-022-18640-8

**Published:** 2022-08-20

**Authors:** JungMin Choi, Sungjae Lee, Mineok Chang, Yeha Lee, Gyu Chul Oh, Hae-Young Lee

**Affiliations:** 1 Department of Internal Medicine, Seoul National University Hospital, Seoul, Republic of Korea; 2 Department of Internal Medicine, Seoul National University College of Medicine, Seoul, Republic of Korea; 3 VUNO Inc, Seoul, Republic of Korea; 4 Division of Cardiology, Department of Internal Medicine, Seoul St. Mary’s Hospital, Seoul, Republic of Korea

**Keywords:** Machine learning, Cardiovascular diseases, Diagnostic markers, Prognostic markers

## Abstract

The performance and clinical implications of the deep learning aided algorithm using electrocardiogram of heart failure (HF) with reduced ejection fraction (DeepECG-HFrEF) were evaluated in patients with acute HF. The DeepECG-HFrEF algorithm was trained to identify left ventricular systolic dysfunction (LVSD), defined by an ejection fraction (EF) < 40%. Symptomatic HF patients admitted at Seoul National University Hospital between 2011 and 2014 were included. The performance of DeepECG-HFrEF was determined using the area under the receiver operating characteristic curve (AUC) values. The 5-year mortality according to DeepECG-HFrEF results was analyzed using the Kaplan–Meier method. A total of 690 patients contributing 18,449 ECGs were included with final 1291 ECGs eligible for the study (mean age 67.8 ± 14.4 years; men, 56%). HFrEF (+) identified an EF < 40% and HFrEF (−) identified EF ≥ 40%. The AUC value was 0.844 for identifying HFrEF among patients with acute symptomatic HF. Those classified as HFrEF (+) showed lower survival rates than HFrEF (−) (log-rank *p* < 0.001). The DeepECG-HFrEF algorithm can discriminate HFrEF in a real-world HF cohort with acceptable performance. HFrEF (+) was associated with higher mortality rates. The DeepECG-HFrEF algorithm may help in identification of LVSD and of patients at risk of worse survival in resource-limited settings.

## Introduction

Left ventricular systolic dysfunction (LVSD) increases the risk of systemic embolism, stroke, and death compared to heart failure (HF) with preserved LV systolic function^1^. Although Vasan et al. showed a decline in asymptomatic LVSD over the past three decades, the prognosis of LVSD has remained unchanged, emphasizing the importance of early diagnosis and adequate management of LVSD^2^. While echocardiography is the standard tool for LVSD diagnosis, the results are highly influenced by operator-dependent factors and its interpretation is subjective, resulting in high dependence to assessor’s expertise^3^. These limitations restrict the routine use of echocardiography in a resource-limited medical setting. Thus, the development of alternative screening tools for LVSD has been attempted, such as biochemical options and electrocardiogram (ECG)^4–9^.

The use of ECG for LVSD diagnosis has been ongoing since 1996, from identification of simple abnormalities on ECG to the more recent development of artificial intelligence (AI) algorithms^5,7–15^. Various AI algorithms have been developed and performed based on different definitions of LVSD (e.g., ejection fraction (EF) < 35%^7,10,14^, < 40%^8,9,11–13^, or < 50%^12^) and for distinct study populations^9,13^. Despite advancement in AI-based LVSD diagnosis, an AI algorithm to identify LVSD patients with an EF < 40% has not been validated in a clinical population of patients with symptomatic HF regardless of EF. To address this gap, we validated the previously developed AI algorithm by Cho et al.^8^ into a deep learning-aided algorithm using ECG for HF with reduced ejection fraction (DeepECG-HFrEF) to identify LVSD, specifically LVSD with an EF < 40% among symptomatic HF patients regardless of EF. For training, we used 12-lead 10 s ECGs recorded from patients with symptomatic HF at Seoul National University Hospital who were enrolled in the Korean Acute HF (KorAHF) Registry. We further evaluated the predictive power of the DeepECG-HFrEF on 5-year all-cause mortality.

## Results

### Baseline characteristics

**Figure 1:**
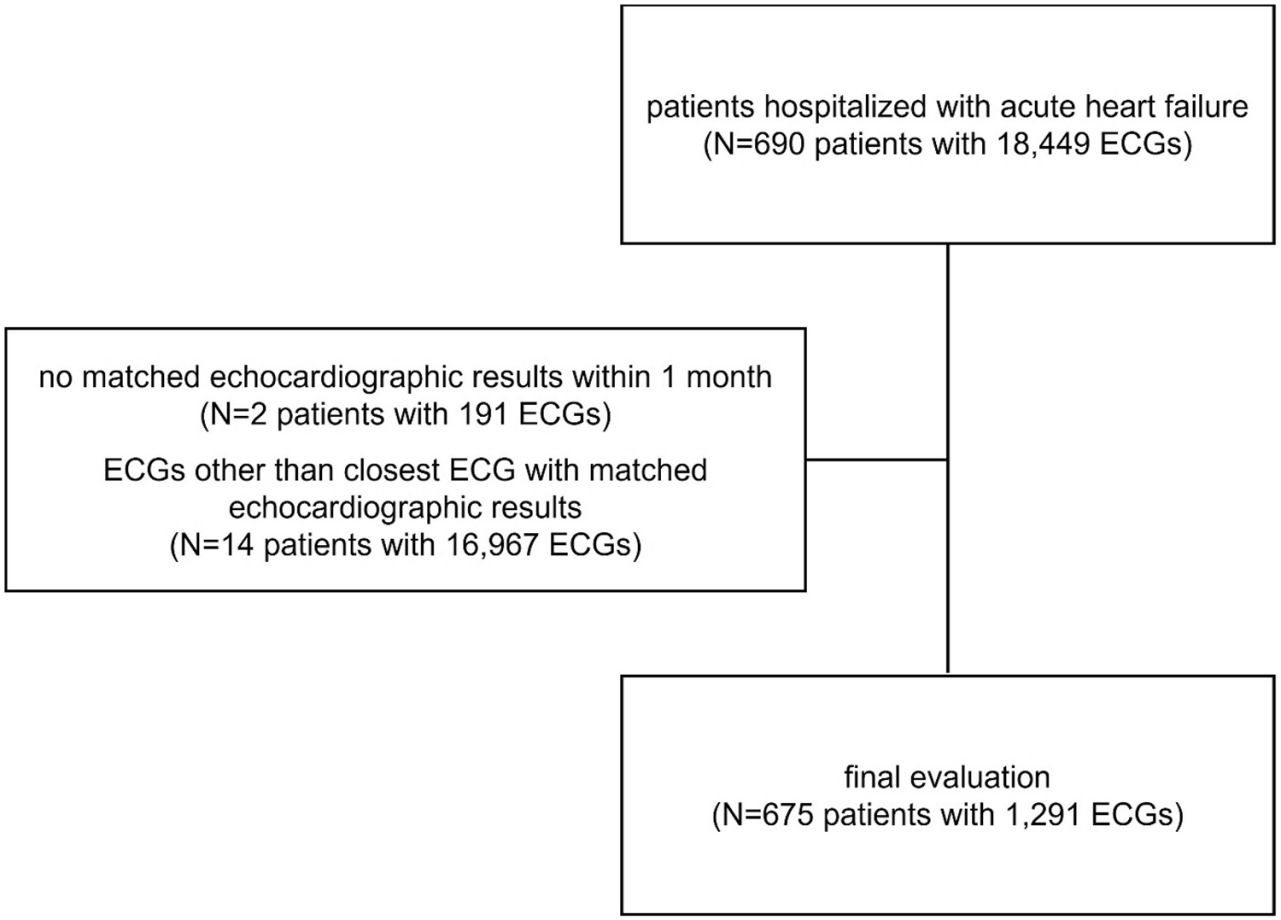
Study flow chart—Among the patients hospitalized with acute heart failure, subjects with no matching echocardiographic results within 1 months and electrocardiograms other than closest match to the echocardiographic results were excluded. *ECG* electrocardiogram.

A total of 690 patients, contributing 18,449 ECGs, who were hospitalized for acute HF were eligible. Of these, those with no matching echocardiography within one month of enrollment (191 ECGs from 2 patients) and ECGs that were not the closest matching to the echocardiography (16,979 ECGs from 14 patients) were excluded. After exclusion, 675 patients contributing 1291 ECGs were included in the analysis (Fig. 1). The mean time interval between the ECG and echocardiography was 29.1 h, with over 82.1% (1060/1291) of the ECGs matched within 24 h of the index echocardiography.

Characteristics of the patients based on the archived ECGs classified by DeepECG-HFrEF algorithm are presented in Table 1. Characteristics of the study population according to echocardiographic results at enrollment are summarized in Supplemental Table S1. Owing to the usage of multiple ECGs from the same patient, the sum of DeepECG-HFrEF based patient-set was larger than the original patient-set. However, the paired datasets were used per patient mostly once or twice. Those classified in the DeepECG-HFrEF (+) group were more likely to be men, to have more comorbidities, to be admitted for de novo HF than for acute decompensated HF, and to present with more severe symptoms of dyspnea. Among the etiologies of HF, ischemic (45.8%) was the most common etiology in the DeepECG-HFrEF (+) group, whereas valvular heart disease (30.1%) was the most common etiology in the DeepECG-HFrEF (−) group. The most prevalent HF group also differed between the two groups, with HFrEF being the most prevalent in the DeepECG-HFrEF (+) group and HFpEF in the DeepECG-HFrEF (−) group. The echocardiographic values differed between the two groups. The DeepECG-HFrEF (+) group showed worse EF along with worse early diastolic velocity (E/e’), and right ventricle systolic pressure (RVSP). These results were consistently observed when confined to ECGs specifically corresponding to HFrEF patients (Supplement Table S2). The confidence score of DeepECG-HFrEF for each ECG was presented with corresponding left ventricular end systolic dimension (LVESD) as scatterplot (Supplement Figure S1). False-positive cases appeared to have smaller LVESD than true-positive cases and similar pattern was seen on false-negative cases when compared to true-negative cases.

**Table 1:** Clinical data of the patients according to the DeepECG-HFrEF algorithm.

	DeepECG-HFrEF (+) (N = 600)	DeepECG-HFrEF (−) (N = 691)	Overall (N = 1291)	*p* value
**Clinical characteristics**				
Age, years	68.5 ± 13.5	67.2 ± 15.2	67.8 ± 14.4	0.102
Men	398 (66.3%)	325 (47.0%)	723 (56.0%)	< 0.001
BMI, kg/m^2^	23.4 ± 4.0	23.7 ± 3.9	23.6 ± 4.0	0.122
Current smoker	90 (15.0%)	88 (12.7%)	178 (13.8%)	0.257
Hypertension	379 (63.2%)	391 (56.6%)	770 (59.6%)	0.017
Diabetes mellitus	266 (44.3%)	237 (34.3%)	503 (39.0%)	< 0.001
Status of HF				0.027
De novo HF	163 (27.2%)	151 (21.9%)	314 (24.3%)	
Acute decompensated HF	437 (72.8%)	437 (78.1%)	977 (75.7%)	
HF class according to EF				< 0.001
HFpEF	90 (15.0%)	448 (64.8%)	538 (41.7%)	
HFmrEF	86 (14.3%)	120 (17.4%)	206 (16.0%)	
HFrEF	424 (70.7%)	123 (17.8%)	547 (42.4%)	
**Echocardiography**				
EF, %	33.5 ± 12.8	51.6 ± 12.9	43.2 ± 15.7	< 0.001
LVESV, mL	131.7 ± 72.2	76.7 ± 46.5	110.7 ± 69.0	< 0.001
LVEDV, mL	184.3 ± 81.9	131.3 ± 55.8	164.1 ± 77.4	< 0.001
E/A ratio	1.6 ± 1.2	1.3 ± 0.9	1.4 ± 1.0	< 0.001
Deceleration time, ms	159.6 ± 66.1	201.0 ± 92.3	182.9 ± 84.4	< 0.001
E/e'	21.9 ± 11.1	19.4 ± 13.0	20.5 ± 12.3	0.001
RVSP, mmHg	48.2 ± 14.7	44.6 ± 15.7	46.2 ± 15.3	< 0.001
**Laboratory**				
eGFR, mL/min/1.73m^2^	57.6 ± 30.7	64.0 ± 32.7	60.9 ± 31.9	0.001
BNP, pg/mL	1745.4 ± 1547.4	1043.4 ± 1240.5	1444.5 ± 1464.5	< 0.001

*BMI* body mass index, *BNP* B-type natriuretic peptide, *ECG* electrocardiogram, *EF* ejection fraction, *eGFR* estimated glomerular filtration rate, *HF* heart failure, *HFpEF* heart failure with preserved ejection fraction, *HFmrEF* heart failure with mid-range ejection fraction, *HFrEF* heart failure with reduced ejection fraction, *LVEDV* left ventricular end-diastolic volume, *LVESV* left ventricular end systolic volume, *RVSP* right ventricular systolic pressure.

### ECG findings

The differences in various ECG parameters between the two DeepECG-HFrEF groups are shown in Table 2. The DeepECG-HFrEF (+) group showed a higher heart rate with longer QRS duration and QTc interval, as well as more prominent QRS widening, QTc prolongation, and Q wave. The two groups did not differ in the PR interval, PR prolongation, or axis. Among causes of QRS widening, left bundle branch block (LBBB) and intraventricular conduction delay (IVCD) were more common in the DeepECG-HFrEF (+) than (−) group (*p* = 0.001). Similar patterns were observed within the ECGs of HFrEF patients (Supplement Table S3).

**Table 2:** ECG findings according to the DeepECG-HFrEF algorithm.

	DeepECG-HFrEF (+) (n = 600)	DeepECG-HFrEF (−) (n = 691)	Overall ECG (n = 1291)	*p* value
Heart rate, pbm	90.7 ± 22.3	79.4 ± 20.5	84.7 ± 22.1	< 0.001
PR interval, ms	173.6 ± 36.5	173.8 ± 40.8	173.7 ± 38.7	0.946
QRS duration, ms	120.3 ± 32.4	106.5 ± 28.5	112.9 ± 31.2	0.049
QTc interval, ms	484.8 ± 48.7	460.1 ± 44.0	471.6 ± 47.8	< 0.001
**Rhythm**				
Sinus rhythm	412 (68.7%)	422 (61.1%)	834 (64.6%)	0.005
AF or AFL	159 (26.5%)	232 (33.6%)	391 (30.3%)	0.006
Other*	30 (5.0%)	37 (5.4%)	67 (5.2%)	0.803
PR prolongation†	62 (16.1%)	57 (14.2%)	119 (9.2%)	0.487
QRS widening‡	134 (22.3%)	94 (13.6%)	228 (17.7%)	< 0.001
LBBB	40 (6.7%)	19 (2.7%)	59 (4.6%)	0.001
RBBB	27 (4.5%)	45 (6.5%)	72 (5.6%)	0.144
IVCD	39 (6.5%)	17 (2.5%)	26 (4.3%)	0.001
QTc prolongation§	440 (73.3%)	317 (45.9%)	757 (58.6%)	< 0.001
Q wave	166 (27.7%)	111 (16.1%)	277 (21.5%)	< 0.001
Anteroseptal	106 (17.7%)	48 (6.9%)	154 (11.9%)	
Lateral wall	11 (1.8%)	1 (0.1%)	12 (0.9%)	
Inferior wall	49 (8.2%)	62 (9.0%)	111 (8.6%)	
Axis				0.937
Normal or LAD	511 (85.3%)	588 (85.1%)	1099 (85.1%)	
RAD or no mans' land	88 (14.7%)	103 (14.9%)	191 (14.8%)	

*VT, VF, high-degree AVB or junctional rhythm.

^†^PR interval > 200 ms.

^‡^QRS duration > 140 ms.

^§^Male > 450 ms, Female > 470 ms.

### Performance of the DeepECG-HFrEF algorithm for different EF cut-offs

The performance of the DeepECG-HFrEF algorithm for different EF cut-off values are reported in Supplement Table S4. Using the optimal cut-off, based on Youden's index, the AUC value for identifying HFrEF among patients with HF was 0.845. For an EF < 40% cut-off, the sensitivity was 0.779, with specificity of 0.763, positive predictive value (PPV) of 0.708, negative predictive value (NPV) of 0.824, and accuracy of 0.770. The AUC, sensitivity, PPV, and accuracy increased, while NPV decreased with an increase in EF.

### Performance of the DeepECG-HFrEF algorithm according to actual EF

**Figure 2:**
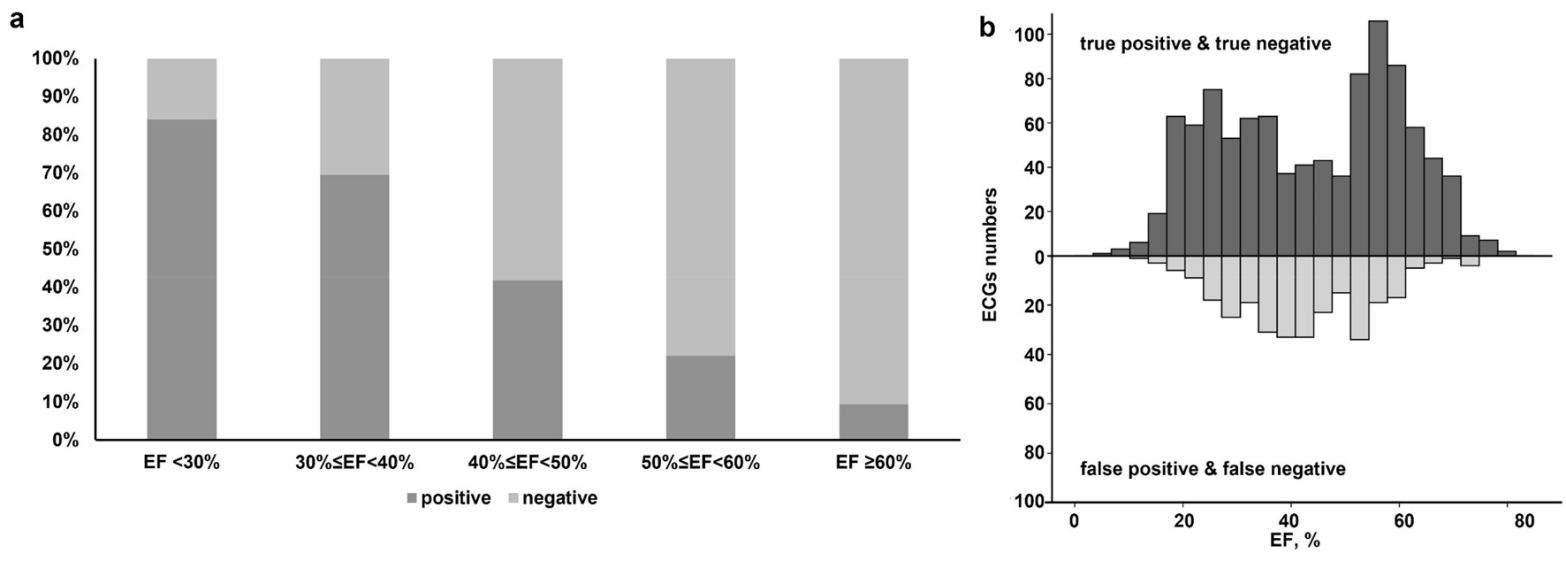
(**a**) Proportion of DeepECG-HFrEF (+) according to actual EF (**b**) Distribution of correct and incorrect cases of DeepECG-HFrEF according to actual EF—The proportion of patients diagnosed with DeepECG-HFrEF (+) increased with the lower actual EF. The false-positives and false-negatives of DeepECG-HFrEF were more likely yielded when the EF was near 40%. *ECG* electrocardiogram, *EF* ejection fraction.

**Figure 3:**
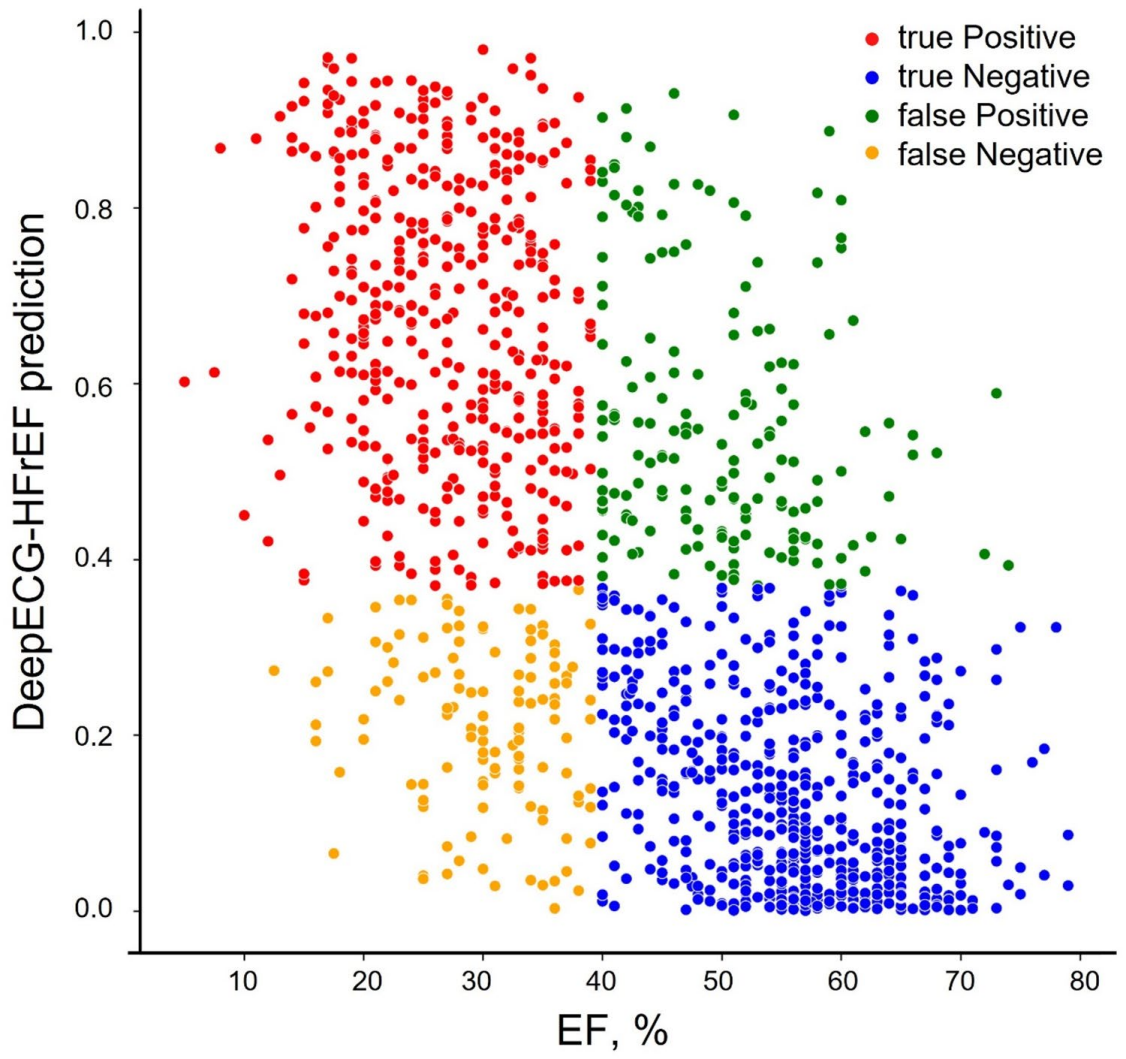
Scatter plot demonstrating observed EF and DeepECG-HFrEF prediction—The proportion of true-positives was higher with lower actual EF even in the scatter plot. *EF* ejection fraction.

The proportion of patients diagnosed with DeepECG-HFrEF (+) increased when the actual EF was lower (Fig. 2A). The DeepECG-HFrEF algorithm was more likely to yield false-positive and false-negative results when the actual EF was near 40% (Fig. 2B). The scatter plot also shows a higher proportion of correct classifications (true-positives) when the actual EF was lower (Fig. 3).

### Performance of DeepECG-HFrEF algorithm in different subpopulations

**Figure 4:**
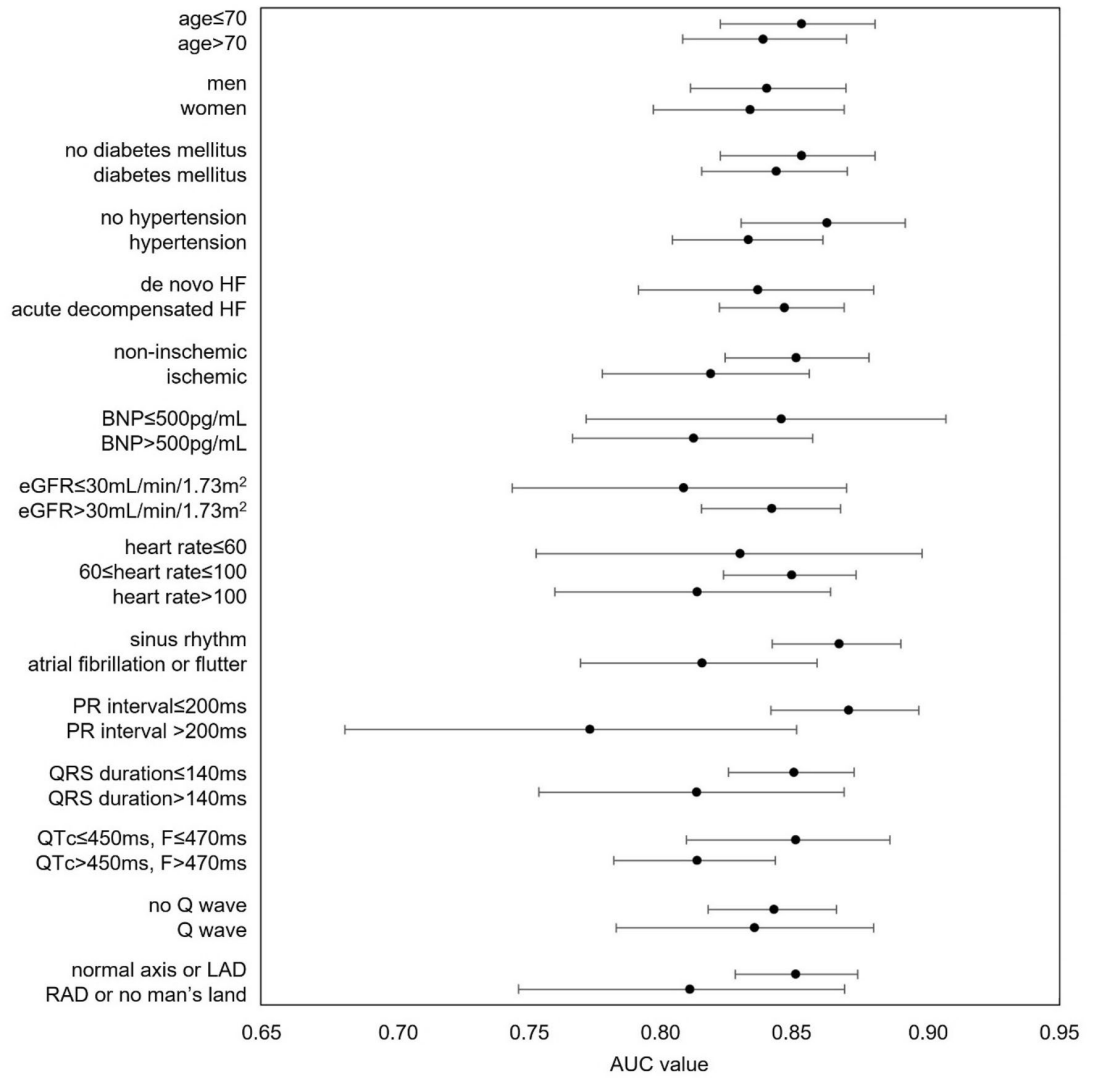
Forest plot depicting AUC values (95% confidence interval) of the DeepECG-HFrEF algorithm in identifying LVSD in different subpopulation**.** The forest plot of DeepECG-HFrEF algorithm showed similar performance among various clinical parameters. *AUC* area under the receiver-operator characteristic curve, *BNP* B-type natriuretic peptide, *EF* ejection fraction, *eGFR* estimated glomerular filtration rate, *HF* heart failure, *LAD* left axis deviation, *RAD* right axis deviation.

Figure 4 is a forest plot of the AUC and associated 95% confidence interval (CI) for the DeepECG-HFrEF algorithm according to various clinical patient parameters. The performance of the DeepECG-HFrEF algorithm was slightly better in the subgroups of patients: age ≤ 70 years, without hypertension, non-ischemic HF, sinus rhythm, PR interval ≤ 200 ms, QRS duration ≤ 140 ms, corrected QT interval of ≤ 450 ms for men and ≤ 470 ms for women, and normal axis or LAD.

### The 5-year all-cause mortality

**Figure 5:**
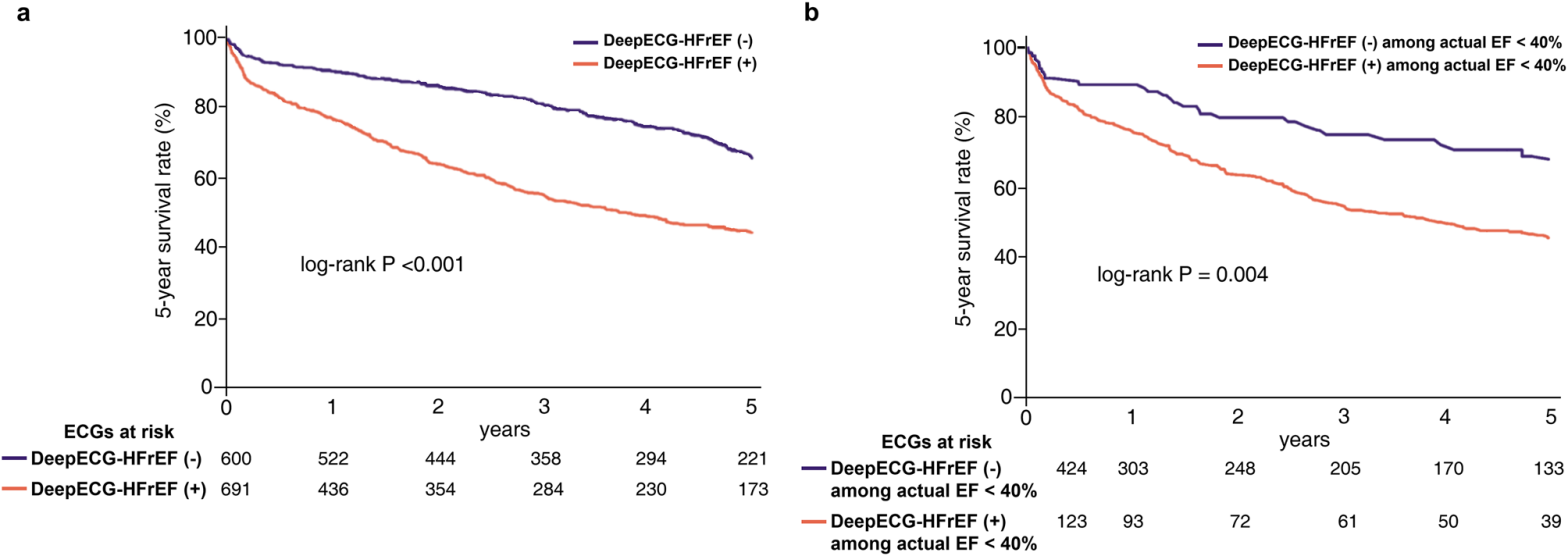
(**a**) Kaplan–Meier curve for mortality at 5-year follow up according to the DeepECG-HFrEF (Total ECGs = 1291) (**b**) Kaplan–Meier curve for mortality at 5-year follow up according to the DeepECG-HFrEF among patients with actual EF < 40%—The patients classified as DeepECG-HFrEF positive showed worse 5-year survival. *ECG* electrocardiogram; *EF* ejection fraction.

Overall, the 5-year survival was worse in the DeepECG-HFrEF (+) than (−) group (*p* < 0.001; Fig. 5A). The Kaplan–Meier curve also showed a lower survival rate among patients with an actual EF< 40% (Fig. 5B). The crude and adjusted hazard ratios (HRs) for 5-year all-cause mortality for the three different models are reported in Table 3 All components of model 1 showed significantly increased crude HR and multivariable-adjusted HR. In model 2, echocardiographic EF < 40% added to model 1, DeepECG-HFrEF (+) remained as significantly higher HR even after multivariable-adjustment. In model 3, which included a B-type natriuretic peptide (BNP) > 500 pg/mL added to model 1, DeepECG-HFrEF (+) was offset by BNP.

**Table 3:** Crude and adjusted hazard ratio for 5-year all-cause mortality among 1291 of heart failure ECGs.

	Crude		Multivariable adjusted	
	HR (95% CI)	*p* value	HR (95% CI)	*p* value
**Model 1**				
Age > 70	2.733 (2.269–3.291)	< 0.001	2.734 (2.192–3.411)	< 0.001
Diabetes	1.674 (1.416–1.980)	< 0.001	1.235 (1.007–1.515)	0.043
Ischemic Heart Disease	1.764 (1.486–2.094)	< 0.001	1.357 (1.106–1.665)	0.003
CKD stage 4–5	1.849 (1.464–2.336)	< 0.001	1.590 (1.250–2.023)	< 0.001
DeepECG-HFrEF (+)	1.496 (1.265–1.770)	< 0.001	1.351 (1.109–1.646)	0.003
**Model 2**				
Age > 70	2.733 (2.269–3.291)	< 0.001	2.726 (2.184–3.403)	< 0.001
Diabetes	1.674 (1.416–1.980)	< 0.001	1.235 (1.006–1.515)	0.043
Ischemic Heart Disease	1.764 (1.486–2.094)	< 0.001	1.362 (1.110–1.673)	0.003
CKD stage 4–5	1.849 (1.464–2.336)	< 0.001	1.591 (1.251–2.023)	< 0.001
DeepECG-HFrEF (+)	1.496 (1.265–1.770)	< 0.001	1.381 (1.099–1.734)	0.006
EF < 40%	1.215 (1.027–1.438)	0.023	1.044 (0.831–1.310)	0.713
**Model 3**				
Age > 70	2.733 (2.269–3.291)	< 0.001	2.693 (1.981–3.660)	< 0.001
Diabetes	1.674 (1.416–1.980)	< 0.001	1.016 (0.770–1.339)	0.913
Ischemic Heart Disease	1.764 (1.486–2.094)	< 0.001	1.375 (1.042–1.813)	0.024
CKD stage 4–5	1.849 (1.464–2.336)	< 0.001	1.719 (1.245–2.375)	0.001
DeepECG-HFrEF (+)	1.496 (1.265–1.770)	< 0.001	1.103 (0.837–1.453)	0.487
BNP > 500, pg/mL	1.693 (1.221–2.348)	0.002	1.585 (1.126–2.232)	0.008

*BNP* B-type natriuretic peptide, *CI* confidence interval, *CKD* chronic kidney disease, *ECG* electrocardiogram, *EF* ejection fraction, *HR* hazard ratio.

## Discussion

In this study, we validated the DeepECG-HFrEF to identify LVSD in patients with symptomatic HF regardless of EF and evaluated the predictive power of the algorithm for the 5-year all-cause mortality. The DeepECG-HFrEF algorithm showed outstanding performance in discriminating LVSD among patients with HF. DeepECG-HFrEF (+) was associated with a worse 5-year survival, even when compared to using the actual EF value. To our knowledge, this is the first study to validate the performance of a deep learning-based AI algorithm for LVSD detection and to show risk predictability in symptomatic patients with HF.

LVSD is identified in 40–50% of patients with HF^16^. Although survival rates of patients with HF have recently improved in developed countries, patients with HF still show an eight-fold higher mortality than an age-matched population^17,18^. Not only does HF increase the risk of mortality, but the associated economic burden cannot be overlooked. The economic burden of HF was estimated to be $108 billion per annum globally in 2012, with 60% direct costs to the healthcare system and 40% indirect costs to society through morbidity and others^19^. Such burden is even higher in Asian countries compared to the United States, with a large proportion of the HF-related healthcare costs directly associated to hospitalization^20^. The impact of this burden is accentuated among elderly patients, with almost three-quarters of the total resources assigned to HF being solely devoted to the older population^21^. The increase in the proportion of elderly individuals in the general population, social ageing phenomenon, is consistent throughout the world, with the elderly population projected to double to almost 1.6 billion globally, from 2025 to 2050^22^. Considering the economic burden of HF in the elderly population, there is a need to improve early diagnosis and treatment of LVSD to slow or even prevent its progression to HF.

A summary of currently developed AI algorithms for the detection of LVSD and the validation of these algorithms is provided in Supplementary Table S5. The definition of LVSD and the primary endpoint differed among studies, with an EF cut-off of 35% to 40% having been used. The study population used for validation also differed between the studies, from using patients at a community general hospital to patients in cardiac intensive care unit and patients with COVID-19^9,12,13^. As a result of these differences in the clinical population used, the proportion of patients within the validation population varied between 2 and 20%^7,11^. Our study is the first to validate the algorithm to detect LVSD solely using patients with HF. Our results showed the strength of the DeepECG-HFrEF algorithm to discriminate LVSD even when the prevalence of HF is high.

Despite recent advances in HF pharmacotherapy, the mortality and rehospitalization rates of patients with HF are still high. Therefore, the identification of high-risk patients who would benefit the most from comprehensive HF treatment is urgently required^23^. A few studies suggested the promising role of AI support for the early diagnosis of low EF^15^. Regarding AI for the detection of LVSD, only one study, by Attia et al., reported on the power of an AI algorithm to predict future LVSD development^7^. Our study is the first to show an association between long-term survival and LVSD of patients with HF based on an AI algorithm. Our results show that the AI algorithm can identify abnormalities in ECG before overt LVSD is observed on echocardiography.

The AI algorithms are known for being a “black box” with exact mechanism unexplainable. However, there are some ECG characteristics in the DeepECG-HFrEF (+) group which might have contributed to the prognostic performance of the algorithm. The DeepECG-HFrEF (+) group had significantly increased corrected QT intervals and increased proportions of LBBB and IVCD. A study by Lee et al. showed that LBBB and IVCD were associated with an increased risk of all-cause mortality and rehospitalization due to HF aggravation^24^. Regarding the QTc interval, a study by Park et al. showed a J-curve association between the corrected QT interval and mortality among patients with acute HF, with a nadir of 440–450 ms in men and 470–480 ms in women^25^. Thus, such an association might be one of the factors used by the DeepECG-HFrEF algorithm to differentiate between the two groups. Nevertheless, as our study did not specifically differentiate the corrected QT interval according to sex, the application of results by Park et al. should be done with caution^25^. Thus, we can carefully interpret that the features shown in the DeepECG-HFrEF (+) group, such as LBBB and IVCD, might be factors that the algorithm is searching for group classification.

There is no clear explanation for the increased false-positive and false-negative rates among patients with an EF near 40%. One plausible explanation might be that the clustering near an EF of 40% may be a heterogeneous group. A previous study by Rastogi et al. showed heterogeneity in the underlying demographics of HFmrEF to be associated with changes in EF over time^26^. Among the HFmrEF groups, improvement in EF tends to be associated with coronary artery disease, while a worsening of EF is more likely to coexist with hypertension and diastolic dysfunction^26^. Patients with acute coronary syndrome are more likely to have dynamic changes in their ECGs and EF over a short period of time^27,28^. As ischemia was the leading cause of acute HF among patients in the KorAHF Registry, such dynamic changes might have contributed to heterogeneity, resulting in a discrepancy between actual EF and DeepECG-HFrEF algorithm results^29^.

### Limitations

The limitations of our study need to be acknowledged in the interpretation of results. First, owing to the retrospective design used, causation between identified factors of LVSD among patients with HF could not be inferred. Further validation of the algorithm using a prospective study design is needed. Second, generalization of our results is limited, and should be cautiously interpreted, as the study population was drawn from a single hospital site in Korea. Further studies on a wider range of race and ethnicity are necessary, as done per the study conducted by the Mayo Clinic using an artificial intelligence-augmented electrocardiogram (AI-ECG) in the United States and Uganda^9,14^. Third, although most of the ECGs were matched to echocardiography within 24 h, some were performed within 30 days. Although these time gaps might influence the performance of our model, the mean ± standard deviation of time gaps for true positive, false positive, false negative, and true negative are 22.0 (± 65.6), 30.6 (± 86.4), 31.3 (± 107.3), and 33.6 (± 90.2), respectively, which was not statistically significant (*p* = 0.192). Also, the performance of the algorithm although the 30-day maximum has generally been accepted in previous studies^10,12^. It is important to note that the ECG matched to echocardiography within 24 h comprised 82.1% of the data used in this study. Fourth, HF medication compliance was not considered. As angiotensin-converting enzyme inhibitors and beta-blockers are known to have a favorable prognosis for the treatment of LVSD, data on such medication adherence would have affected survival. Fifth, our study focused on the association between ECG and echocardiography and included multiple ECG and echocardiographic data from one person. This may have had a slight influence on the survival analysis. A sequential study using a single ECG and echocardiography from individual patients would be useful to confirm our results. Lastly, our study used visually estimated EF values documented by the examiners because EF measurement by Simpson’s biplane or other calculated methods were inadequate either by poor echocardiographic window or severely unbalanced myocardial contraction (61 out of 1291 cases).

## Conclusions

The DeepECG-HFrEF algorithm showed acceptable performance in distinguishing HFrEF in a real-world HF cohort. Patients with a DeepECG-HFrEF (+) classification had a significantly worse 5-year survival. Application of the DeepECG-HFrEF algorithm may be of specific benefit in resource-limited clinical settings where echocardiography is not readily eligible to identify high-risk patients who may benefit from active therapeutic intervention.

## Methods

### Statement of ethics

Our Institutional Review Boards approved this retrospective database study at Seoul National University Hospital (No.2012-191-1186). The requirement for informed consent from the study subjects was waived by the IRB of Seoul National University Hospital due to the retrospective study design. All research was performed in accordance with the Declaration of Helsinki. Use of the data from the KorAHF Registry was previously approved (Institutional Review Boards of Seoul National University Hospital No. 2004-166-1119)^29^.

### Study population

This was a retrospective validation study of the AI ECG algorithm for patients with symptomatic HF at Seoul National University Hospital. The ECGs used to validate the DeepECG-HFrEF for the diagnosis of HFrEF were retrieved from the KorAHF Registry. Eligible were patients who had undergone ECG and echocardiography within a 30-day interval. Patients with missing demographics, ECGs, and echocardiographic information were excluded. For patients who underwent repeated ECGs and echocardiography, all records were verified, and the ECGs performed closest to (before or after) the index echocardiography selected for analysis. All ECGs included in the analysis were manually reviewed by two certified cardiologists to confirm the cardiac rhythm diagnosis.

### Data management

Demographic and echocardiographic data, and clinical outcomes were obtained from the KorAHF Registry^25,29^. The 12-lead ECGs were performed using the MUSE system (MAC 5500 HD, versions 5D to 8, GE Healthcare), at a sampling rate of 500 Hz. The left ventricular EF was determined using the following hierarchical approach: Simpson’s biplane method was used preferentially; if this was not available, then other calculated methods were used; and finally, if EF could not be calculated, then visual estimation was used. HF was classified according to the left ventricular EF, as follows: HFrEF (EF < 40%); HF with mildly reduced EF (HFmrEF, EF: 40–50%); and HF with preserved EF (HFpEF, EF > 50%)^30^.

### AI Algorithm

The original convolutional neural network (CNN)-based algorithm was previously described, developed, and externally validated^8^. The DeepECG-HFrEF algorithm to detect a LVEF < 40% was validated to detect an EF < 40% from 12-lead 10 s ECGs data of HF patients. The algorithm was implemented on the TensorFlow (Google, Mountain View, CA) framework and written in Python (version 3.6; Python Software Foundation, Beaverton, OR). For this study, the algorithm was newly implemented on PyTorch (Facebook, Menlo Park, CA), with no additional training or optimization of the original algorithm. The output for the algorithm is a continuous value between 0 and 1, representing a confidence score for an EF < 40%. Using a certain cut-off value, all tests either had a positive (+) or negative (−) result, and none of the tests were considered intermediate.

### Statistical analysis

A comprehensive panel of diagnostic performance metrics was summarized to evaluate the performance of the DeepECG-HFrEF algorithm. In particular, the sensitivity, specificity, PPV, NPV, accuracy, and accuracy of the validation study were determined using the original algorithm positive (+) of greater than or equal to the cut-off of 0.370, indicating that the input ECG had a confidence score of 0.370 to detect a LVEF < 40%^8^. The AUC with confidence interval was evaluated via a 2000-sample bootstrapping method. We examined the optimal threshold, which is defined as the threshold that maximizes the sum of sensitivity and specificity (i.e., Youden’s index). Continuous variables are presented as the mean ± standard deviation and compared using the unpaired Student’s t-test. Categorical variables were expressed as frequencies or percentages and were compared using the chi-squared test. For the secondary objective of exploring the long-term prognostic impact of DeepECG-HFrEF (+), the Kaplan–Meier method was used with between-group differences assessed using the log-rank test. The Cox proportional-hazards regression model was used to identify the predictors of 5-year all-cause mortality. The performance of three models was evaluated: DeepECG-HFrEF (+) model 1 (age > 70 years, diabetes, ischemic heart disease, and chronic kidney disease (CKD) stage 4–5); DeepECG-HFrEF (+) model 2 (echocardiographic results of EF < 40%, age > 70 years, diabetes, ischemic heart disease, and CKD stage 4–5); and DeepECG-HFrEF (+) model 3 (BNP > 500 pg/mL, age > 70 years, diabetes, ischemic heart disease, and CKD stage 4–5). All reported *p*-values were two-sided, with a *p*-value < 0.05 considered significant. Statistical analyses were performed using IBM SPSS Statistics version 23 (IBM Co., Armonk, NY, USA).

## Supplementary Information

Supplementary Information.

## Data Availability

The datasets used and/or analyzed during the current study are all available from the corresponding author on reasonable request.
