# Author Correction: Deep learning of ECG waveforms for diagnosis of heart failure with a reduced left ventricular ejection fraction

**DOI:** 10.1038/s41598-022-22012-7

**Published:** 2022-10-13

**Authors:** JungMin Choi, Sungjae Lee, Mineok Chang, Yeha Lee, Gyu Chul Oh, Hae-Young Lee

**Affiliations:** 1 Department of Internal Medicine, Seoul National University Hospital, Seoul, Republic of Korea; 2 Department of Internal Medicine, Seoul National University College of Medicine, Seoul, Republic of Korea; 3 VUNO Inc, Seoul, Republic of Korea; 4 Division of Cardiology, Department of Internal Medicine, Seoul St. Mary’s Hospital, Seoul, Republic of Korea

Correction to: *Scientific Reports* https://doi.org/10.1038/s41598-022-18640-8, published online 20 August 2022

The original version of this Article contained an error in Figure 5, where the label of the orange-colored line in panel (b) was incorrectly given as "DeepECG HFrEF (-) among EF < 40%". The original Figure 5 and accompanying legend appear below.

**Figure 5:**
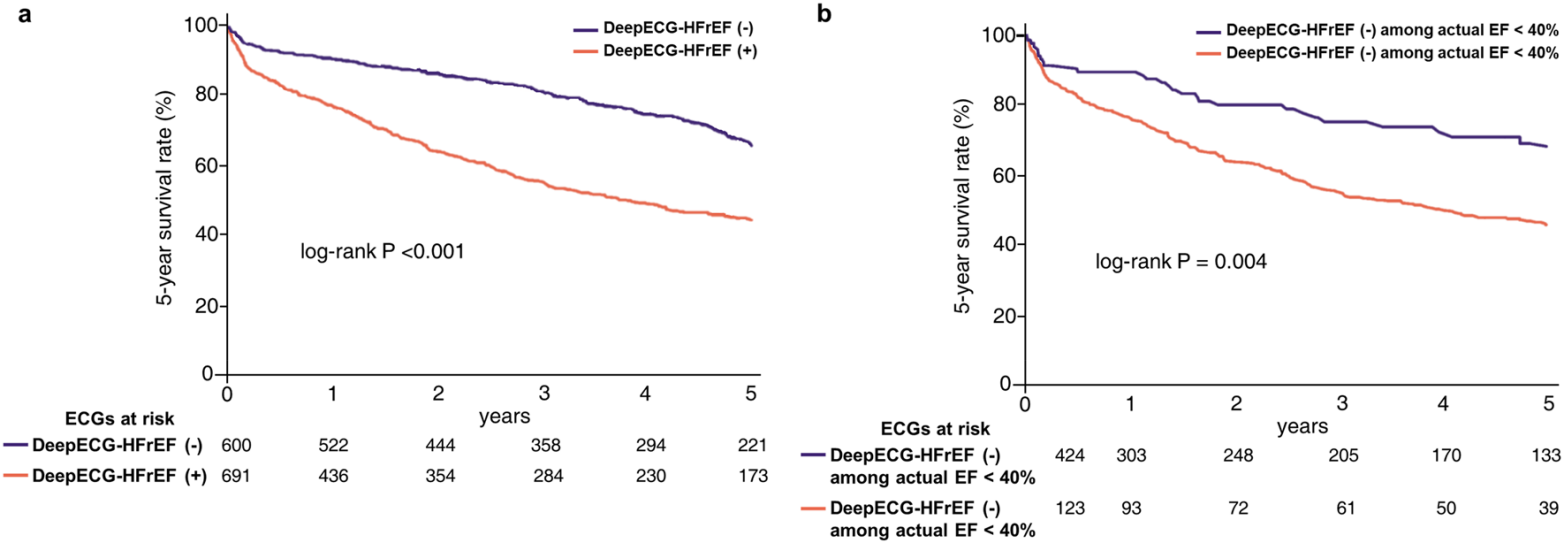
(**a**) Kaplan–Meier curve for mortality at 5-year follow up according to the DeepECG-HFrEF (Total ECGs = 1291) (**b**) Kaplan–Meier curve for mortality at 5-year follow up according to the DeepECG-HFrEF among patients with actual EF < 40%—The patients classified as DeepECG-HFrEF positive showed worse 5-year survival. *ECG* electrocardiogram; *EF* ejection fraction.

The original Article has been corrected.

