# Supplementary material for: Deep learning of ECG waveforms for diagnosis of heart failure with a reduced left ventricular ejection fraction
Source: Sci Rep. 2022 Aug 20;12:14235. doi: 10.1038/s41598-022-18640-8 (PMC9392508; doi:10.1038/s41598-022-18640-8)
Supplement: Supplementary file 1 — Supplementary Information. [file 41598_2022_18640_MOESM1_ESM.docx]

**Supplement**

**Supplementary Figure S1.** Scatterplot demonstrating LVESD and the performance of DeepECG-HFrEF algorithm for identifying LVSD

**Supplementary Tables**

**Table S1.** Baseline characteristics of study population according to the first echocardiographic results

**Table S2.** Clinical data of ECGs corresponding to HFrEF classified by the DeepECG-HFrEF algorithm

**Table S3.** Characteristics of ECGs corresponding to HFrEF classified by the DeepECG-HFrEF algorithm

**Table S4.** Performance of DeepECG-HFrEF algorithm using different EF cut-offs

**Table S5.** Studies on validation of AI algorithms developed for identification of LVSD

**Figure S1. Scatterplot demonstrating LVESD and the performance of DeepECG-HFrEF algorithm for identifying LVSD**

**
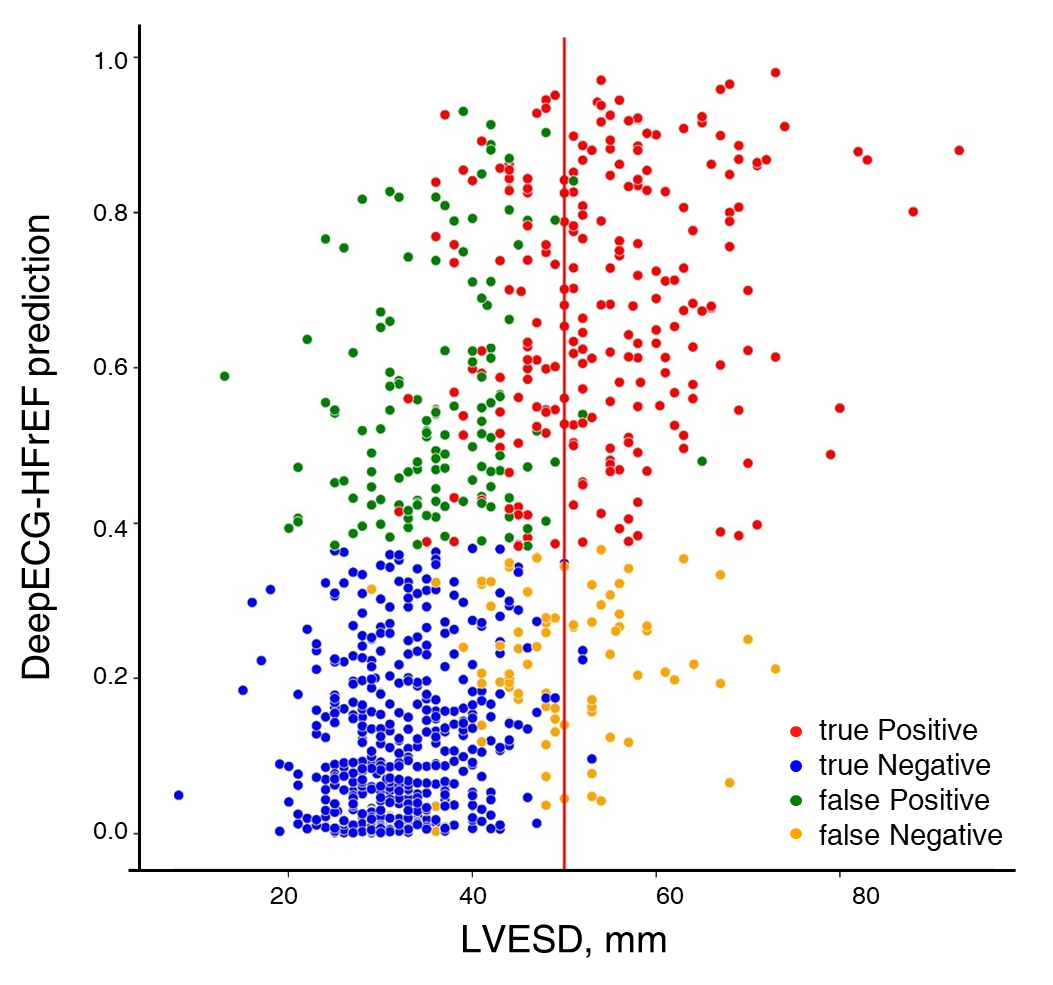
**

Abbreviation: LVESD, left ventricular end systolic dimension; LVSD, left ventricular systolic dysfunction.

**Table S1. Baseline characteristics of study population according to the first echocardiographic results**

|  | **HFrEF (N=349)** | **HFmrEF (N=113)** | **HFpEF (N=213)** | **p value** |
| --- | --- | --- | --- | --- |
| **Clinical characteristics** |  |  |  |  |
| Age, years | 66.5±14.5 | 72.0±11.8 | 68.5±15.1 | 0.002 |
| Men | 237 (67.9%) | 56 (49.6%) | 90(42.3%) | <0.001 |
| BMI, kg/m^2^ | 23.3±4.2 | 23.3±3.5 | 23.7±4.1 | 0.480 |
| Current smoker | 59(16.9%) | 11(9.7%) | 24(11.3%) | 0.064 |
| Hypertension | 207(59.3%) | 40(35.4%) | 129(60.6%) | 0.698 |
| Diabetes mellitus | 150(43.0%) | 40(35.4%) | 70(32.9%) | 0.043 |
| Status of HF |  |  |  | 0.309 |
| De novo HF | 153(43.8%) | 58(51.3%) | 103(48.4%) |  |
| Acute decompensated HF | 196(56.2%) | 55(48.7%) | 110(51.6%) |  |
| NYHA Class IV | 210(60.2%) | 70(61.9) | 94(44.1%) | <0.001 |
| Etiology of HF |  |  |  | <0.001 |
| Ischemic heart disease | 161(46.1%) | 50(44.2%) | 17(22.1%) |  |
| Valvular heart disease | 31(8.9%) | 15(13.3%) | 88(41.3%) |  |
| Cardiomyopathy | 123(35.2%) | 28(24.8%) | 36(16.9%) |  |
| Other | 34(9.7%) | 20(17.7%) | 42(19.7%) |  |
| Counts of paired dataset per patient | | | | 0.557 |
| Once | 133(38.1%) | 45(39.8%) | 77(36.2%) |  |
| Two times | 130(37.2%) | 47(41.6%) | 77(36.2%) |  |
| Three times | 71(20.3%) | 19(16.8%) | 46(21.6%) |  |
| Four times | 15(4.3%) | 2(1.8%) | 13(6.1%) |  |
| **Echocardiography** |  |  |  |  |
| EF, % | 27.1±7.2 | 44.2±3.0 | 59.3±6.3 | <0.001 |
| LVESV, mL | 143.7±72.9 | 65.9±23.3 | 54.7±35.6 | <0.001 |
| LVEDV, mL | 193.4±85.4 | 117.3±39.7 | 118.9±55.8 | <0.001 |
| E/A ratio | 1.8±1.2 | 1.2±0.9 | 1.3±1.0 | <0.001 |
| Deceleration time, ms | 145.8±64.0 | 177.6±84.3 | 185.6±68.1 | <0.001 |
| E/e' | 22.1±13.2 | 18.1±9.8 | 19.0±10.7 | 0.004 |
| RVSP, mmHg | 48.5±13.6 | 44.9±14.2 | 49.8±17.9 | 0.037 |
| **Laboratory** |  |  |  |  |
| eGFR, mL/min/1.73m^2^ | 59.8±32.9 | 62.8±31.2 | 65.0±31.8 | 0.176 |
| BNP, pg/mL | 1796.9±1579.5 | 1203.7±1244.9 | 1019.8±1229.1 | <0.001 |

Abbreviation: BMI, body mass index; BNP, B-type natriuretic peptide; EF, ejection fraction; eGFR, estimated glomerular filtration rate; HF, heart failure; HFpEF, heart failure with preserved ejection fraction; HFmrEF, heart failure with mid-range ejection fraction; HFrEF, heart failure with reduced ejection fraction; NYHA, New York Heart Association; LVEDV, left ventricular end-diastolic volume; LVESV, left ventricular end systolic volume; RVSP, right ventricular systolic pressure.

**Table S2. Clinical data of ECGs corresponding to HFrEF classified by the DeepECG-HFrEF algorithm**

|  | **DeepECG-HFrEF (+) N=424** | **DeepECG-HFrEF (-) N=123** | **Overall ECG  N=547** | **p value** |
| --- | --- | --- | --- | --- |
| **Clinical characteristics** | | | | |
| Age | 68.2±13.7 | 68.2±13.3 | 68.2±13.6 | 0.984 |
| Male | 300(70.8%) | 71(57.7%) | 371(67.8%) | 0.008 |
| BMI, kg/m^2^ | 23.5±4.0 | 23.2±4.0 | 23.4±4.0 | 0.436 |
| Current smoker | 74(17.5%) | 20(16.3%) | 94(17.2%) | 0.892 |
| Hypertension | 261(61.6%) | 76(61.8%) | 337(61.6%) | 1.000 |
| Diabetes | 197(46.5%) | 50(40.7%) | 247(45.2%) | 0.260 |
| Status of HF |  |  |  | 0.362 |
| De novo HF | 123(29.0%) | 30(24.4%) | 153(28.0%) |  |
| Acute decompensated HF | 301(71.0%) | 93(75.6%) | 394(72.0%) |  |
| **Echocardiography** | | | | |
| EF, % | 26.6±6.9 | 30.1±6.1 | 27.4±6.9 | <0.001 |
| LVESV, mL | 150.0±70.2 | 114.6±55.8 | 142.3±68.8 | <0.001 |
| LVEDV, mL | 201.1±81.3 | 163.7±69.9 | 193.0±80.4 | <0.001 |
| E/A ratio | 1.7±1.2 | 1.3±0.9 | 1.6±1.2 | 0.007 |
| Deceleration time, ms | 145.7±50.5 | 175.4±104.0 | 152.9±68.7 | 0.008 |
| E/e' | 22.7±11.4 | 20.3±16.9 | 22.1±12.9 | 0.096 |
| RVSP, mmHg | 48.4±13.8 | 44.0±13.8 | 47.4±13.9 | 0.005 |
| **Laboratory** | | | | |
| eGFR, 30mL/min/1.73m2 | 57.7±30.9 | 58.5±31.3 | 57.9±30.9 | 0.818 |
| BNP, ng/L | 1904.1±1636.5 | 1227.0±1131.4 | 1785.2±1579.0 | 0.001 |

Abbreviation: BMI, body mass index; BNP, B-type natriuretic peptide; EF, ejection fraction; eGFR, estimated glomerular filtration rate; HF, heart failure; HFpEF, heart failure with preserved ejection fraction; HFmrEF, heart failure with mid-range ejection fraction; HFrEF, heart failure with reduced ejection fraction; LVEDV, left ventricular end-diastolic volume; LVESV, left ventricular end systolic volume; RVSP, right ventricular systolic pressure.

**Table S3. Characteristics of ECGs corresponding to HFrEF classified by the DeepECG-HFrEF algorithm**

|  | **DeepECG-HFrEF (+) N=424** | **DeepECG-HFrEF (-) N=123** | **Overall ECG**  **N=547** | **p value** |
| --- | --- | --- | --- | --- |
| Heart rate, bpm | 91.9±21.6 | 82.5±24.7 | 89.8±22.6 | <0.001 |
| PR interval, ms | 172.1±36.6 | 176.0±37.4 | 172.9±36.8 | 0.420 |
| QRS duration, ms | 123.2±33.4 | 123.3±38.2 | 123.2±34.5 | 0.990 |
| QTc interval, ms | 488.1±47.6 | 480.5±49.4 | 486.4±48.1 | 0.121 |
| Rhythm |  |  |  |  |
| Sinus rhythm | 310(73.1%) | 74(60.2%) | 384(70.2%) | 0.007 |
| AF or AFL | 95(22.4%) | 37(30.1%) | 132(24.1%) | 0.094 |
| Other* | 20(4.7%) | 12(9.8%) | 32(5.9%) | 0.048 |
| PR prolongation† | 37(13.0%) | 12(16.4%) | 49(9.0%) | 0.448 |
| QRS widening‡ | 111(26.2%) | 39(31.7%) | 150(27.4%) | 0.251 |
| LBBB | 32(7.5%) | 12(9.8%) | 44(8.0%) | 0.452 |
| RBBB | 22(5.2%) | 11(8.9%) | 33(6.0%) | 0.134 |
| IVCD | 32(7.5%) | 7(5.7%) | 39(7.1%) | 0.556 |
| QTc prolongation§ | 327(77.1%) | 85(69.1%) | 412(75.3%) | 0.075 |
| Q wave | 126(29.7%) | 27(22.0%) | 153(28.0%) | 0.110 |
| anteroseptal | 78(18.4%) | 14(11.4%) | 92(16.8%) |  |
| Lateral wall | 9(2.1%) | 1(0.8%) | 10(1.8%) |  |
| inferior wall | 39(9.2%) | 12(9.8%) | 51(9.3%) |  |
| Axis |  |  |  | 0.161 |
| Normal or LAD | 361(85.3%) | 98(79.7%) | 459(83.9%) |  |
| RAD or no man's land | 62(14.7%) | 25(20.3%) | 87(15.9%) |  |

*VT, VF, high-degree AVB or junctional rhythm. †PR interval >200ms. ‡QRS duration >140ms. §Male >450ms, Female >470ms

Abbreviation: AF, atrial fibrillation; AFL, atrial flutter; IVCD, intraventricular conduction delay; LAD, left axis deviation; LBBB, left bundle branch block; RAD, right axis deviation; RBBB, right bundle branch block.

**Table S4. Performance of DeepECG-HFrEF algorithm using different EF cut-offs**

| **EF** | **Prevalence** | **AUC (95% CI)** | **Sensitivity** | **Specificity** | **PPV** | **NPV** | **Accuracy** |
| --- | --- | --- | --- | --- | --- | --- | --- |
| <50% | 0.583 | 0.850 (0.829-0.871) | 0.811 | 0.723 | 0.804 | 0.733 | 0.775 |
| <40% | 0.424 | 0.845 (0.823-0.866) | 0.779 | 0.763 | 0.708 | 0.824 | 0.770 |
| <30% | 0.235 | 0.816 (0.790-0.840) | 0.760 | 0.739 | 0.472 | 0.909 | 0.744 |

Abbreviation: AUC, Area under the receiver operating curve; CI, confidence interval; EF, ejection fraction; PPV, positive predictive value; NPV, negative predictive value.

**Table S5. Studies on validation of AI algorithms developed for identification of LVSD**

| **Study** | **Location** | **Network** | **Name of AI algorithm** | **Endpoint LVSD definition** | **Study population^a^** | **ECG^a^, N** | **Men^a^,**  **N (%)** | **Age^a^,**  **(Mean/Median Years)** | **HF^a^,  N (%)** | **AUC** |
| --- | --- | --- | --- | --- | --- | --- | --- | --- | --- | --- |
| **Attia et al.(1),**  **Retrospective** | USA | CNN | — | EF ≤ 35 | — | 8,989 | 5,168 (57%) | 61.8 ± 16.5^a^ | 1,803 (20%) | 0.93^b^ |
| **Kwon et al.(2),**  **Retrospective** | Korea | DNN | DEHF | EF ≤ 40 | Community general hospital^a^ | 9,965^c^  10,490^d^ | 11,907 (52%) | — | 1391 (6%) ^a^ | 0.84^c^  0.89^d^ |
| **Adedinsewo et al.(3), Retrospective** | USA | CNN | AI-ECG | EF ≤ 35 | Patients presenting to the emergency department with acute dyspnea | — | 847 (53%) | 67.9 | — | 0.89 |
| **Mondo et al.(4), Retrospective** | Uganda | CNN | AI-ECG | EF ≤ 35 | General cardiac clinic | — | 266 (42%) | 57.0 ± 18.8 | 77 (12%) | 0.87 |
| **Attia et al.(5), Retrospective** | USA | CNN | AI-ECG | EF ≤ 40 | COVID-19 patients | — | 18 (67%) | 66.5 ± 13.8 | — | 0.95 |
| **Cho et al.(6), Retrospective** | Korea | CNN | — | EF < 40 | Cardiovascular teaching hospital^a^ | 3,470^c^  4,362^d^ | 2610^d^ (60%) | — | 2,021 (12%) | 0.91^c^  0.96^d^ |
| **Jentzer et al.(7), Retrospective** | USA | CNN | AI-ECG | EF ≤ 40 | Cardiac intensive care unit patients | — | 3588 (63%) | 68.2 ± 14.8 | 960 (17%) | 0.83 |
| **Kashou et al.(8), Retrospective** | USA | CNN | AI-ECG | EF ≤ 40 | Community-based cohort | — | 983 (48%) | 63 ± 11 | 45 (2%) | 0.97 |

— Not specifically mentioned in the paper

^a^ Validation set ^b^ Test set ^c^ Internal validation ^d^ External validation

Abbreviation: AI, artificial intelligence; AI-ECG, artificial intelligence-enabled ECG; AUC, area under the receiver operating characteristic; CNN, convolutional neural network; DEHF, deep-learning algorithm for ECG-based HF identification; DNN, deep neural network; ECG, electrocardiogram; EF, ejection fraction; HF, heart failure; LVSD, left ventricular systolic dysfunction; USA, United States of America.

**References**

1. Attia ZI, Kapa S, Lopez-Jimenez F, McKie PM, Ladewig DJ, Satam G, et al. Screening for cardiac contractile dysfunction using an artificial intelligence–enabled electrocardiogram. Nature Medicine. 2019;25(1):70-4.

2. Kwon J-m, Kim K-H, Jeon K-H, Kim HM, Kim MJ, Lim S-M, et al. Development and Validation of Deep-Learning Algorithm for Electrocardiography-Based Heart Failure Identification. kcj. 2019;49(7):629-39.

3. Adedinsewo D, Carter RE, Attia Z, Johnson P, Kashou AH, Dugan JL, et al. Artificial Intelligence-Enabled ECG Algorithm to Identify Patients With Left Ventricular Systolic Dysfunction Presenting to the Emergency Department With Dyspnea. Circ Arrhythm Electrophysiol. 2020;13(8):e008437.

4. Mondo CK, Attia ZI, Benavente ED, Friedman P, Noseworthy P, Kapa P, et al. External validation of an electrocardiography artificial intelligence-generated algorithm to detect left ventricular systolic function in a general cardiac clinic in Uganda. European Heart Journal. 2020;41(Supplement_2).

5. Attia ZI, Kapa S, Noseworthy PA, Lopez-Jimenez F, Friedman PA. Artificial Intelligence ECG to Detect Left Ventricular Dysfunction in COVID-19: A Case Series. Mayo Clinic Proceedings. 2020;95(11):2464-6.

6. Cho J, Lee B, Kwon JM, Lee Y, Park H, Oh BH, et al. Artificial Intelligence Algorithm for Screening Heart Failure with Reduced Ejection Fraction Using Electrocardiography. Asaio j. 2021;67(3):314-21.

7. Jentzer JC, Kashou AH, Attia ZI, Lopez-Jimenez F, Kapa S, Friedman PA, et al. Left ventricular systolic dysfunction identification using artificial intelligence-augmented electrocardiogram in cardiac intensive care unit patients. International Journal of Cardiology. 2021;326:114-23.

8. Kashou AH, Medina-Inojosa JR, Noseworthy PA, Rodeheffer RJ, Lopez-Jimenez F, Attia IZ, et al. Artificial Intelligence-Augmented Electrocardiogram Detection of Left Ventricular Systolic Dysfunction in the General Population. Mayo Clin Proc. 2021;96(10):2576-86.
